# Bird’s Eye View of Emerging Zoonoses

**DOI:** 10.3201/eid1602.AC1602

**Published:** 2010-02

**Authors:** Polyxeni Potter

**Affiliations:** Centers for Disease Control and Prevention, Atlanta, Georgia, USA

**Keywords:** Ellis Wilson, art science connection, emerging infectious diseases, art and medicine, Caribbean Bird Vendor, zoonoses, about the cover

**Figure:**
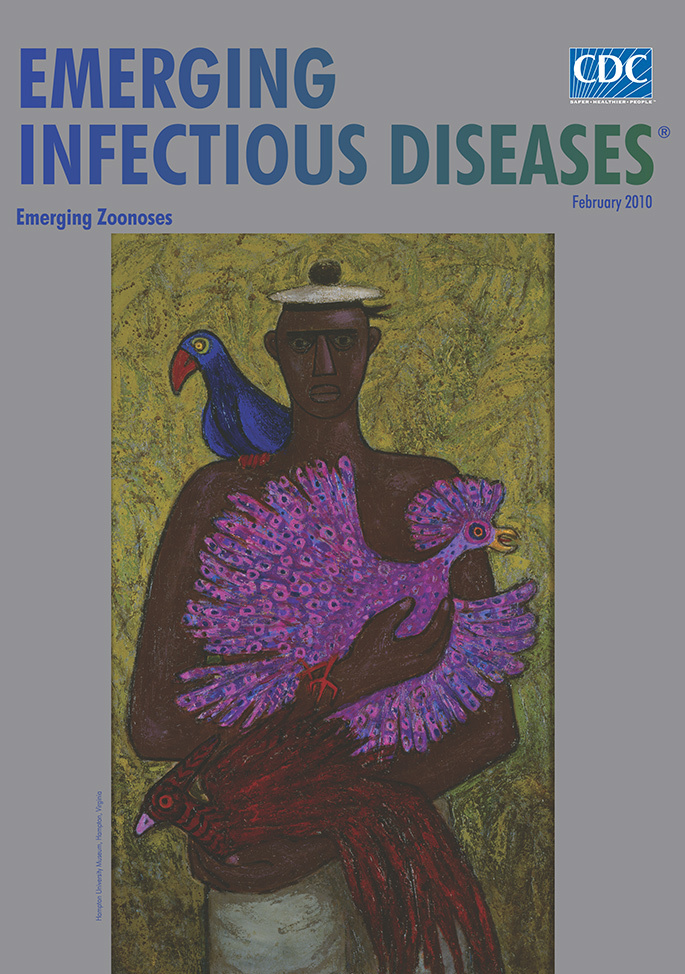
**Ellis Wilson (1899–1977) Caribbean Bird Vendor (1953)** Oil on canvas (91.44 cm × 60.96 cm) Hampton University Museum, Hampton Virginia

“So much to paint and so little time” was Ellis Wilson’s assessment of his chosen profession. “I want to paint all the time―everything of interest and beauty.” His approach was to recreate life around him, people at work. He painted them making turpentine, mining clay, harvesting tobacco, buying and selling goods. His portrait of an aircraft-engine factory worker in New Jersey won him a Guggenheim Fellowship in 1944. But his best known painting is probably Funeral Procession, a work displayed above the mantle of the Huxtables’ living room in the 1980s–90s television series The Cosby Show.

Wilson was born in Mayfield, a cloth-manufacturing town in Kentucky’s tobacco-growing region. The son of a barber-cabinet maker who took art lessons and painted with some success, he was inspired by his father to study art from a young age. “I guess I got what little talent I have from him,” he said in a 1975 interview. Young Wilson did odd jobs and worked as janitor at Day’s Ready-to-Wear, where his weekly portraits drawn with cleaning wax on the dress shop window delighted passersby.

The aspiring artist attended Kentucky State College in Frankfort, then moved north to continue his studies at the Art Institute of Chicago in 1919, a period of racial upheaval and riots. “I couldn’t go downtown to the Art Institute. They were shooting and carrying on.” He joined the Chicago Art League and met many in the art community, among them sculptor Richmond Barthé. “I had never been in a group of artists …. It was just great to be numbered among them.” After completing his studies, he worked as commercial artist for a few years, then traveled to New York in 1928, during the period of cultural and artistic resurgence Harlem Renaissance.

In New York, Wilson broke away from the academic style taught him in Chicago and from representational painting to focus on shape and color. His forms became increasingly angular and elongated, the colors brighter and more vibrant, the faces spare and masklike with a modernist inflection. “I just cut out completely from anything that looked like a portrait. It was freer. I was astounded.” He worked for the Federal Art Project sponsored by the Works Progress Administration and at various other jobs, joined the Harlem Artists Guild, and continued to paint and meet other painters: Aaron Douglas, Alain Locke, Joseph Delaney, Palmer Hayden. He moved to Greenwich Village, where he exhibited at Augusta Savage’s Salon and other venues.

“Practically all my life I have been painting under difficult conditions,” Wilson said of earning a living from art. “Lack of money and time, especially time, has prevented me from painting as much and as often as I have wanted to.” Dealers “wanted all black painters to be ‘primitives,’” and the public viewed him with mistrust. “A white woman who bought pictures from me invited me to her home in North Carolina. I told inquisitive people that I did painting for her in New York. They thought I was a house painter.”

Despite these difficulties, Wilson sought and received fellowships, awards, and other opportunities, which allowed him to roam the southern states, the Sea Islands, and later Haiti. The experiences broadened his artistic scope and nourished his creativity. “I could express myself to the fullest degree and accomplish worthwhile work.” In Charleston, South Carolina, he became fascinated with the local open market, its vendors and crowds and the hustle and bustle of their daily activities. He observed and sketched them freely and marveled at the authenticity and honesty of their lives. “I noticed such great hopes among the people in the South: hopes that they could soon vote, and hopes that education would become free and open. My own hope is that I capture their hopes in my work.”

His work in Haiti, marked by exuberant color and figures free of extraneous detail, was similarly expansive, “I’d never seen a tropical place―and with the music, the drumming, the dancing… [the people] were very artistic.” This work was well received back in New York, where he returned to live and paint until his death. But his legacy as an innovator, both in his choice of subject matter and geometric boldly colored form, was not fully appreciated until later.

“I hold my honey and I store my bread / In little jars and cabinets of my will / I label clearly, and each latch and lid / I bid, Be firm till I return from hell,” wrote Gendolyn Brooks (1917–2000) in her poem “My Dreams, My Works, Must Wait Till after Hell,” expressing the frustrations of her generation and giving words to Wilson’s dilemma. All his training, high profile works, contacts in the art world, all his talent and dedication could not overcome the odds against him. He died unknown and was buried in an unmarked, “pauper’s” grave. Fewer than 100 of his paintings have been located.

Caribbean Bird Vendor, on this month’s cover, captures Wilson’s love of beauty as a main goal in art. The flat fully delineated form suits the regal figure he has placed against yellowing vegetation and directly in front of the viewer. Sturdy and solid, this vendor is sketched with little embellishment or detail, yet his geometric features denote sadness, and he seems preoccupied. His eyes, unfocused, show detachment from his circumstances as a seller of birds. The parrot on his shoulder is perched with ease, claws withdrawn. The bird in the lower right is also at peace, nesting attentively in his cupped palm. But the one in the center is panic stricken. Pulling in the opposite direction from the other birds, its feathers ruffled and spread, beak open, eye glaring, claws flexing, it is ready for flight. The vendor seems ambivalent as he steadies it on his chest.

The vibrant colors only add to the tension in this scene. The bird in distress, the most attractive and volatile, draws all the attention, its frantic state amplifying the unnatural calm of the vendor, who seems not controlling but protective, as if uncomfortable with the intent to sell his charges. A means to earn a living, the sale of birds and other wildlife out of their natural habitat is part of economic reality in much of the world. This vendor may well loathe his own occupation.

Apart from the cruelty inherent in animal displacement and captivity, sensed by Wilson’s vendor, other reasons, not least of them public health, compel against the sale and import of exotic birds. Unsanitary conditions in live bird markets in the United States and abroad have come under scrutiny in recent years, when legal and illegal trade in domestic and wild birds was associated with the global spread of highly pathogenic avian influenza (H5N1). This issue of Emerging Infectious Diseases features other health crises associated with animals on the move, from leptospirosis to Marburg virus infection and white-nose syndrome in bats, and examines fungal infections that could spread through inhaling dust contaminated with spores from bird droppings.

As he painted other people at work, Wilson was sensitive to the socioeconomic factors that prevented him from achieving greatness in his own time. What he did not know was that the same factors interfere with health, in a web of zoonotic and environmental interconnections.
